# Supplementary material for: Profiling neuronal methylome and hydroxymethylome of opioid use disorder in the human orbitofrontal cortex
Source: Nat Commun. 2023 Jul 28;14:4544. doi: 10.1038/s41467-023-40285-y (PMC10382503; doi:10.1038/s41467-023-40285-y)
Supplement: Supplementary file 1 — Supplementary Information [file 41467_2023_40285_MOESM1_ESM.pdf]

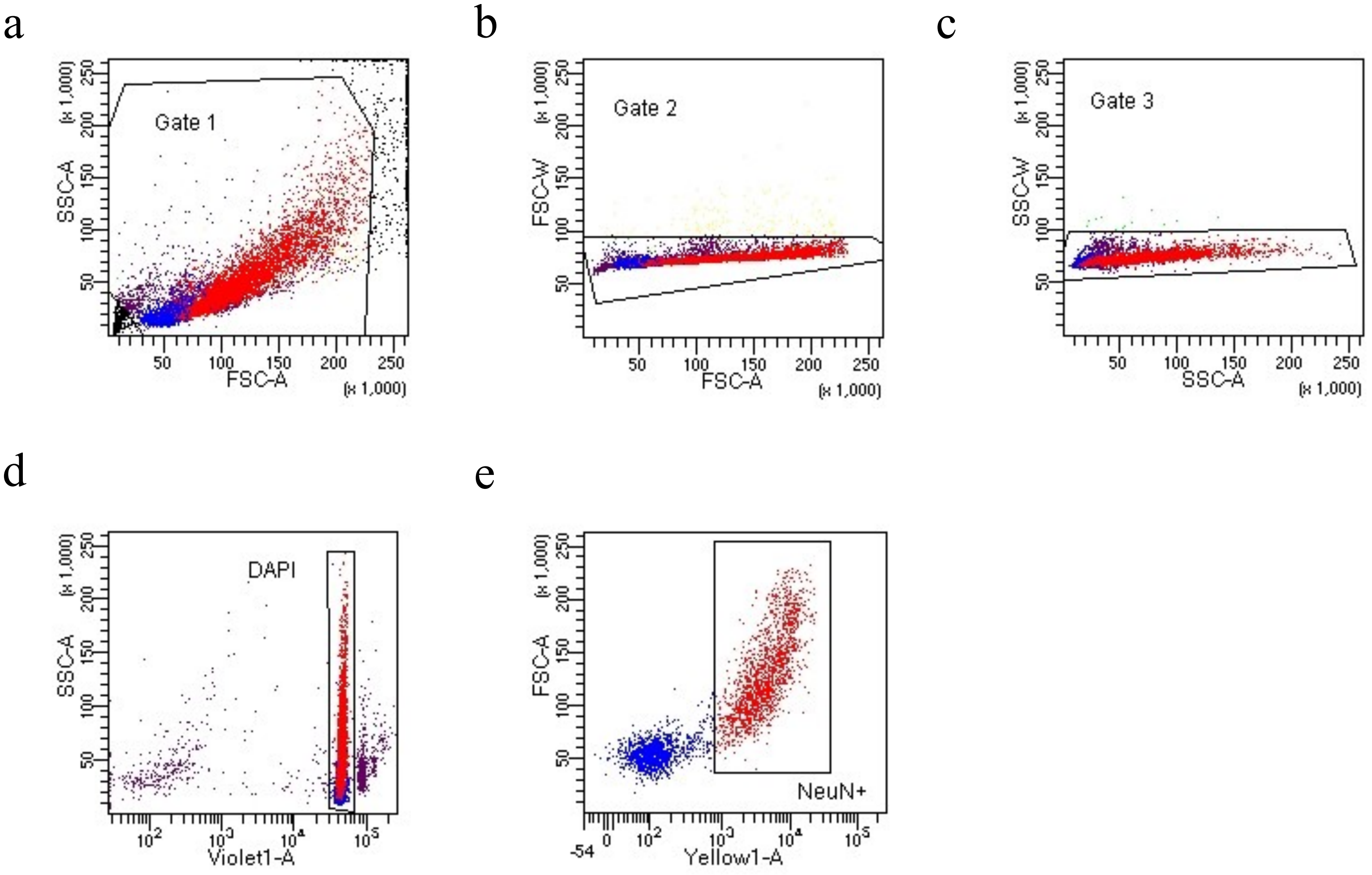

f

Tube: Representative sample

Population	#Events	%Parent	%Total
All Events	10,000	####	100.0
Gate 1	9,035	90.4	90.4
Gate 2	8,886	98.4	88.9
Gate 3	8,871	99.8	88.7
DAPI	8,038	90.6	80.4
NeuN+	4,382	54.5	43.8

Supplementary Figure 1. Representative FANS gating. FANS of single nuclei. Nuclei stained with NeuN-PE conjugated antibody were filtered through a 40-µm cell strainer and loaded onto a custom FACS ARIA II flow sorter (Becton Dickinson) equipped with a forward scatter photomultiplier tube. a) Particles smaller than nuclei were first eliminated with an area plot of forward scatter (FSC-A) vs. side scatter (SSC-A). b-c) Plots of area vs. width in the forward and side scatter channels, respectively, are used for doublet discrimination with gating to exclude aggregates of 2 or more nuclei. d) Remaining nuclei were then gated in the violet wavelength while excluding the remaining doublet and triplet signals. e) The final gating panel captured NeuN+ neuronal nuclei using the yellow emission spectra. NeuN+ nuclei represented ~50-60% of DAPI+ nuclei. f) Summary of the representative samples observed in the plots a-e.

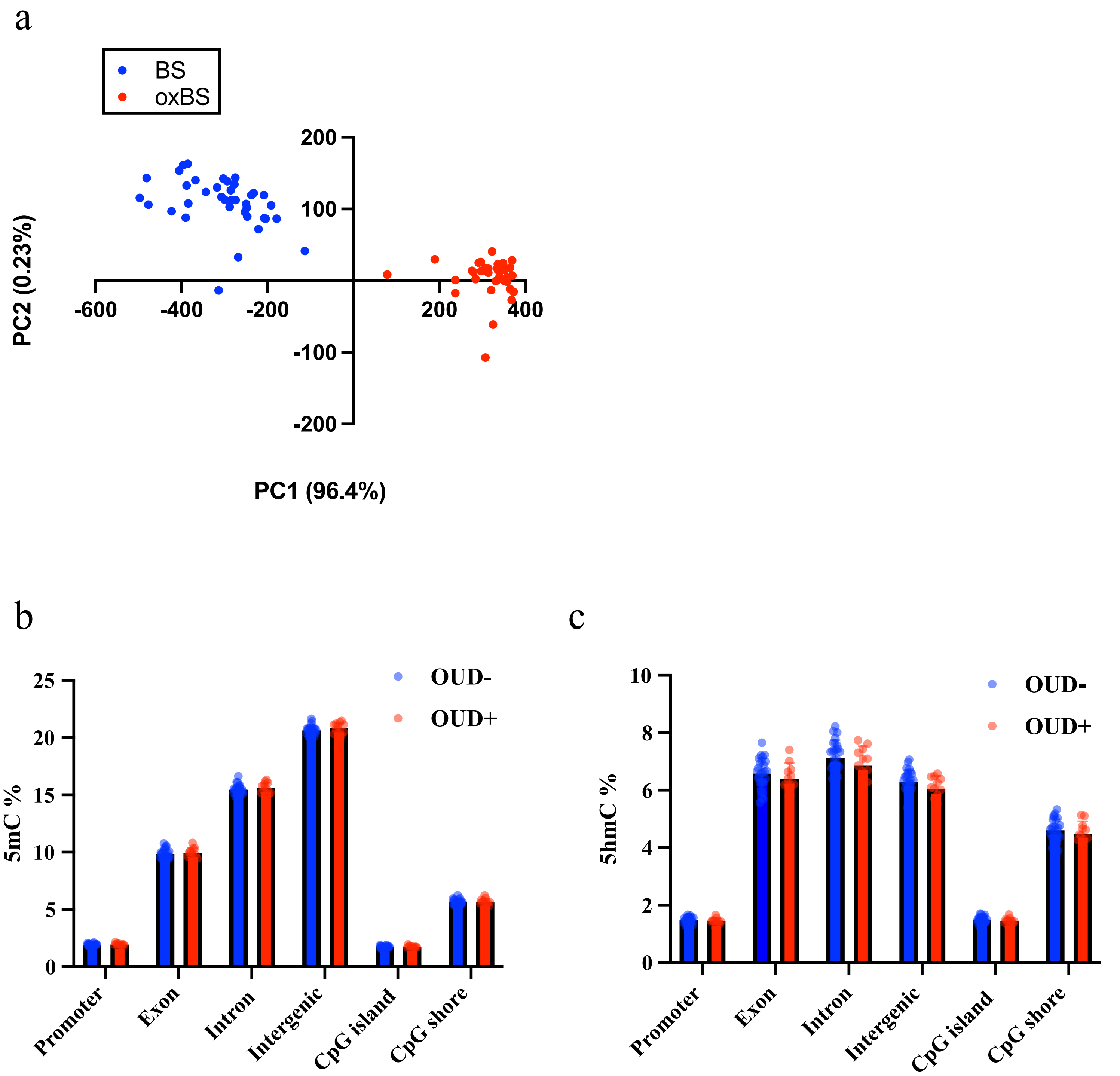

Supplementary Figure 2. a) Principal component analysis to identify outliers. b) Distribution of 5mC CpGs in the gene region (n=38 biologically independent samples). c) Distribution of 5hmC CpGs in the gene region (n=26 biologically independent samples). . In b and c, Data are presented as mean values +/- SD.

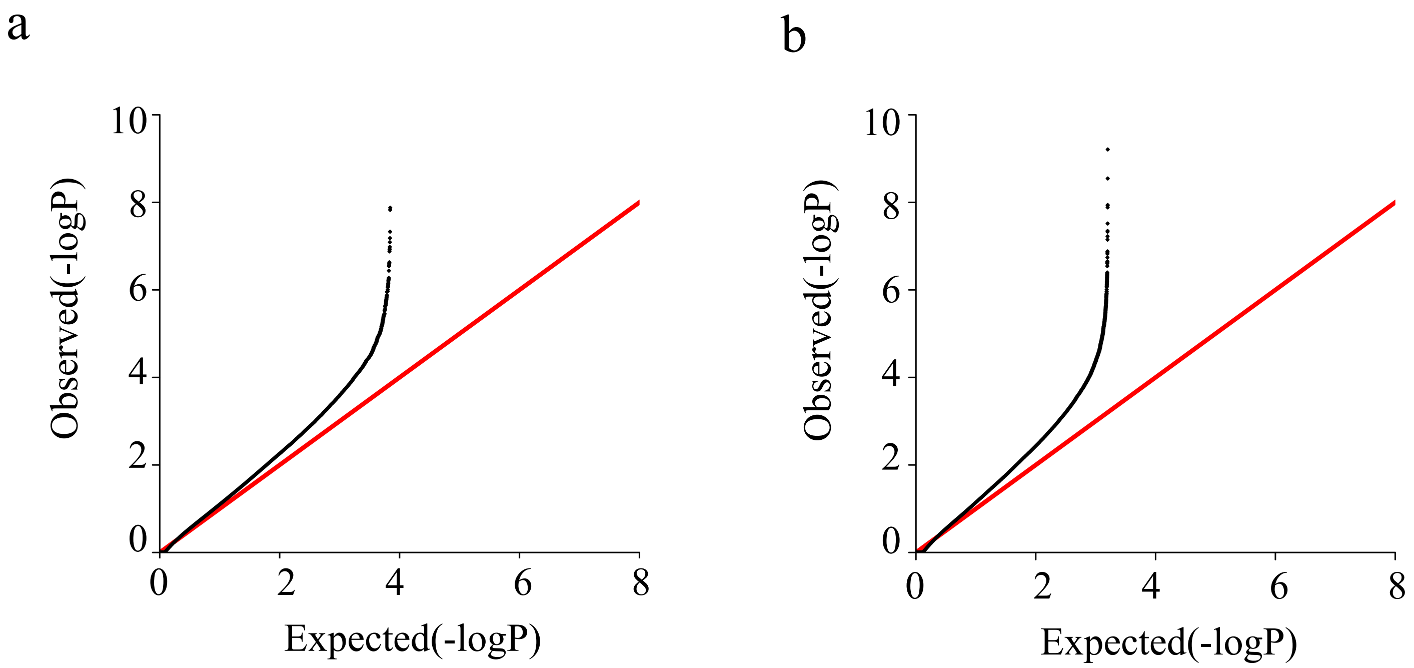

Supplementary Figure 3. QQ-plots. a) 5mC differential analysis; b) 5hmC differential analysis. QQ plots were calculated using a probability distribution of the data.

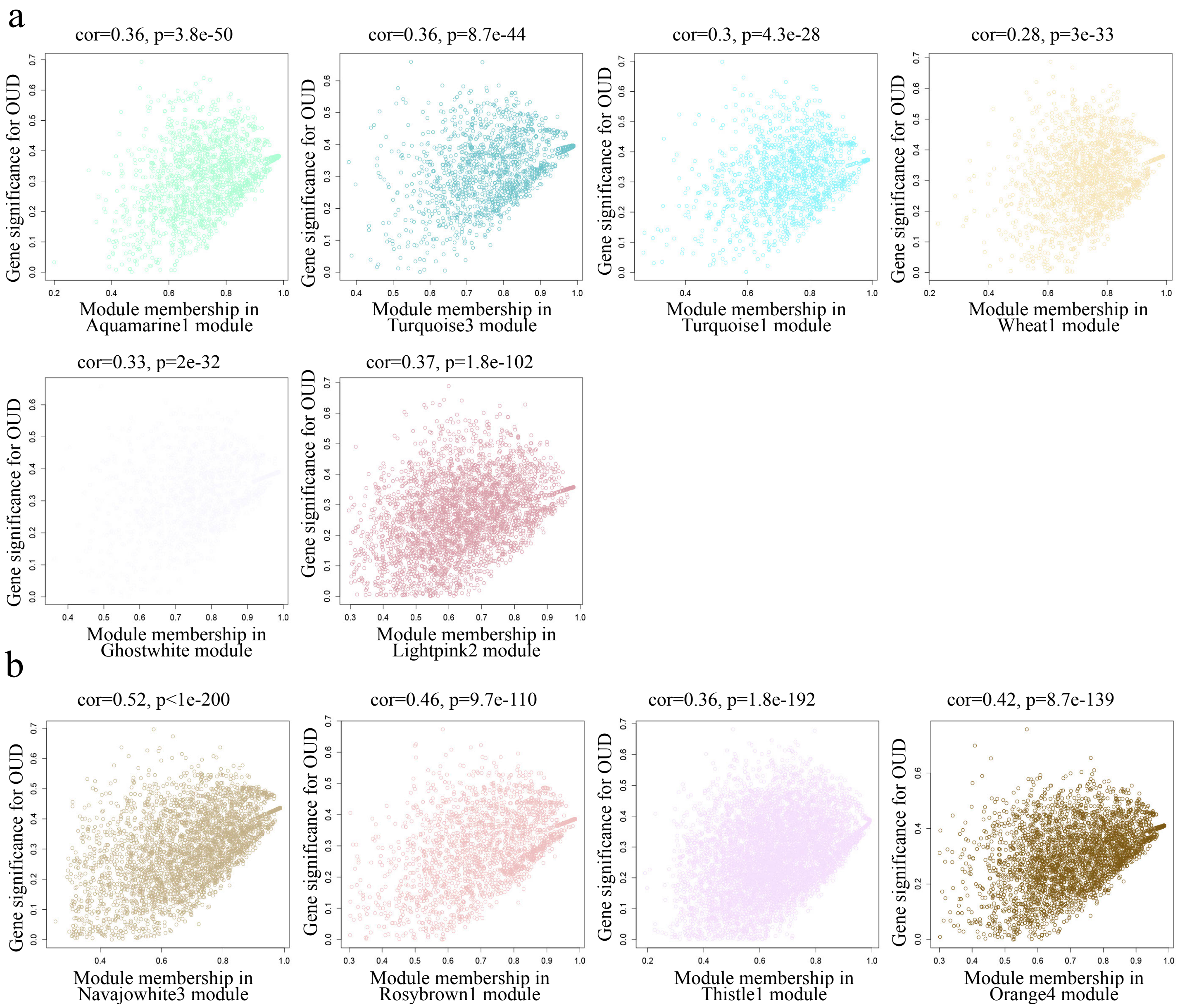

Supplementary Figure 4. Exploring 5mC and 5hmC co-methylation results. a) Module membership vs. gene significance in 5mC co-methylation analysis. b) Module membership vs. gene significance in 5hmC co-methylation analysis. The correlations were calculated using weighted Pearson correlation, and p-value using student asymptotic statistics.

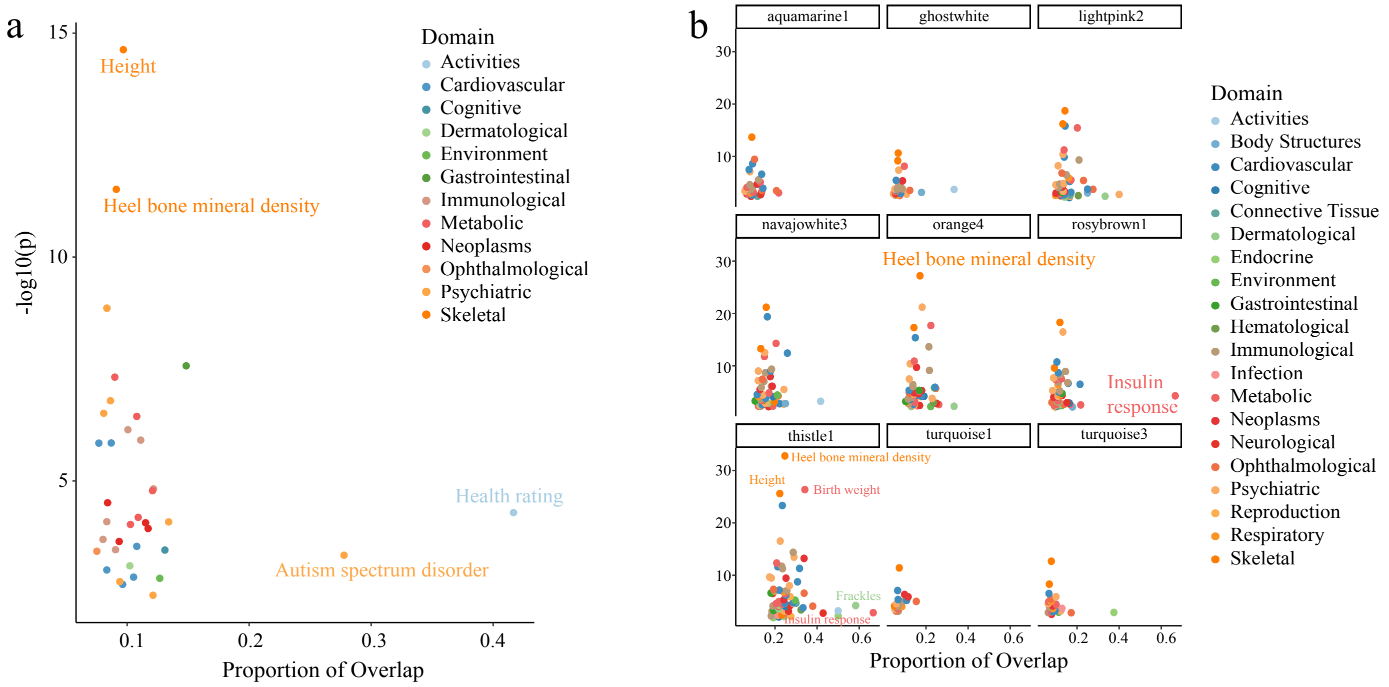

Supplementary Figure 5. Genome-wide association enrichment signals analysis. a) GWAS enrichment analysis of the annotated genes for 5hmC differential CpGs. b) GWAS enrichment analysis for co-methylated and co-hydroxymethylated modules. Enrichment analyses were performed using Fisher exact test.
