# Supplementary material for: Profiling neuronal methylome and hydroxymethylome of opioid use disorder in the human orbitofrontal cortex
Source: Nat Commun. 2023 Jul 28;14:4544. doi: 10.1038/s41467-023-40285-y (PMC10382503; doi:10.1038/s41467-023-40285-y)
Supplement: Supplementary file 3 — Description of Additional Supplementary Files [file 41467_2023_40285_MOESM3_ESM.pdf]

Description of Additional Supplementary Files

File Name: Supplementary Data 1
Description: **Differential CpG Methylation (FDR-adjusted q < 0.05).** Differential analyses were performed using methylkit, which applies logistic regression to calculate the p-values.

File Name: Supplementary Data 2
Description: **Differential CpG Hydroxymethylation (FDR-adjusted q < 0.05).** Differential analyses were performed using methylkit, which applies logistic regression to calculate the p-values.

File Name: Supplementary Data 3
Description: **Genomic Region and Feature Enrichment Analysis.** Enrichment analysis was performed using the Fisher's exact test.

File Name: Supplementary Data 4
Description: **Gene ontology enrichment of the differentially methylated genes.** Enrichment analysis was performed using the Fisher's exact test.

File Name: Supplementary Data 5
Description: **Gene ontology enrichment of the differentially hydroxymethylated genes.** Enrichment analysis was performed using the Fisher's exact test.

File Name: Supplementary Data 6
Description: **Overlapping differentially methylated genes between OUD and heroin overdose.** The table shows all the annotated genes of the differential 5mC and 5hmC sites that overlapped with the heroin overdose study of Kozlenkov et al, 2017.

File Name: Supplementary Data 7
Description: **Gene ontology enrichment of the overlapped differentially methylated genes between OUD and heroin overdose.** Enrichment analysis was performed using the Fisher's exact test.

File Name: Supplementary Data 8
Description: **Literature overview.** The table shows the differential 5mC and 5hmC CpG sites previously associated with opioid-related traits, considering 5mC at CpG sites.

File Name: Supplementary Data 9
Description: **Drug interaction analysis of genes with differential 5mC in OUD.** The table shows all the drug interactions described for the annotated genes with differential 5mC.

File Name: Supplementary Data 10
Description: **Drug interaction analysis of genes with differential 5hmC in OUD.** The table shows all the drug interactions described for the annotated genes with differential 5hmC.

File Name: Supplementary Data 11
Description: **Gene ontology enrichment of the 5mC co-methylation modules.** Enrichment analysis was performed using the Fisher's exact test.

File Name: Supplementary Data 12
Description: **Gene ontology enrichment of the 5hmC co-methylation modules.** Enrichment analysis was performed using the Fisher's exact test.

File Name: Supplementary Data 13
Description: **Reactome pathways associated with the 5mC and 5hmC modules.** Enrichment analysis was performed using the Fisher's exact test.

File Name: Supplementary Data 14
Description: **GWAS enrichment analysis of the differential 5hmC genes associated with OUD.** GWAS enrichment analysis was performed using the hypergeometric test.

File Name: Supplementary Data 15
Description: **GWAS enrichment analysis of the co-methylated and co-hydroxymethylated modules.** GWAS **enrichment** analysis was performed using the hypergeometric test.

File Name: Supplementary Data 16
Description: **Differential expression analysis of OUD.** The differential expression analysis was performed using DESeq2, which applies a generalized linear model to calculate the log2FC.

File Name: Supplementary Data 17
Description: **Correlation analysis between 5mC and gene expression.** The correlation analysis was performed using Pearson correlation.

File Name: Supplementary Data 18
Description: **Correlation analysis between 5mC and gene expression.** The correlation analysis was performed using Pearson correlation.
