# Supplementary material for: Profiling neuronal methylome and hydroxymethylome of opioid use disorder in the human orbitofrontal cortex
Source: Nat Commun. 2023 Jul 28;14:4544. doi: 10.1038/s41467-023-40285-y (PMC10382503; doi:10.1038/s41467-023-40285-y)
Supplement: Supplementary file 5 — Reporting Summary [file 41467_2023_40285_MOESM5_ESM.pdf]

nature portfolio

Corresponding author(s): Janitza L. Montalvo-Ortiz, Ph.D.

Last updated by author(s): 2023/05/22

Reporting Summary

Nature Portfolio wishes to improve the reproducibility of the work that we publish. This form provides structure for consistency and transparency in reporting. For further information on Nature Portfolio policies, see our Editorial Policies and the Editorial Policy Checklist.

Statistics

For all statistical analyses, confirm that the following items are present in the figure legend, table legend, main text, or Methods section.

n/a	Confirmed	
☐	☒	The exact sample size (*n*) for each experimental group/condition, given as a discrete number and unit of measurement
☒	☐	A statement on whether measurements were taken from distinct samples or whether the same sample was measured repeatedly
☐	☒	The statistical test(s) used AND whether they are one- or two-sided<br>*Only common tests should be described solely by name; describe more complex techniques in the Methods section.*
☐	☒	A description of all covariates tested
☐	☒	A description of any assumptions or corrections, such as tests of normality and adjustment for multiple comparisons
☐	☒	A full description of the statistical parameters including central tendency (e.g. means) or other basic estimates (e.g. regression coefficient) AND variation (e.g. standard deviation) or associated estimates of uncertainty (e.g. confidence intervals)
☐	☒	For null hypothesis testing, the test statistic (e.g. *F*, *t*, *r*) with confidence intervals, effect sizes, degrees of freedom and *P* value noted<br>*Give P values as exact values whenever suitable.*
☒	☐	For Bayesian analysis, information on the choice of priors and Markov chain Monte Carlo settings
☒	☐	For hierarchical and complex designs, identification of the appropriate level for tests and full reporting of outcomes
☐	☒	Estimates of effect sizes (e.g. Cohen's *d*, Pearson's *r*), indicating how they were calculated

Our web collection on statistics for biologists contains articles on many of the points above.

Software and code

Policy information about availability of computer code

Data collection	No software was used for data collection
Data analysis	Bioinformatic analysis: Bismark bisulfite read mapper (v0.15.0), methylkit R package (v1.10.0), methylGSA Bioconductor package (v.3.5), Genomation R package (v.1.4.2), biomaRt R package (v.2.28.0), UCSC genome browser (GenGen software tools 2010Apr29 version; hg38.knownGene.gtf 2020-01-10 09:34, hg38.refGene.gtf 2020-01-10 09:33), WGCNA R package (1.69), AmiGO (v.2.5), STRING (v.11.0b), DESeq2 package in R (1.34.0), MatrixEQTL package (v.2.3), FUMA (v1.3.6b), David (v2021q4), Drug Gene Interaction Database (DGIdb; v4.2.0).

For manuscripts utilizing custom algorithms or software that are central to the research but not yet described in published literature, software must be made available to editors and reviewers. We strongly encourage code deposition in a community repository (e.g. GitHub). See the Nature Portfolio guidelines for submitting code & software for further information.

Data

Policy information about availability of data

All manuscripts must include a data availability statement. This statement should provide the following information, where applicable:

- Accession codes, unique identifiers, or web links for publicly available datasets
- A description of any restrictions on data availability
- For clinical datasets or third party data, please ensure that the statement adheres to our policy

For this work we have used the Human Genome (GRCh38), for annotation we have used the Homo_sapiens.GRCh38.84.gtf.

The source data and summary statistics data generated in this study are provided in the Supplementary Information file. The beta values data generated in this study have been deposited in the GitHub database under DOI: 10.5281/zenodo.7958290.

Research involving human participants, their data, or biological material

Policy information about studies with human participants or human data. See also policy information about sex, gender (identity/presentation), and sexual orientation and race, ethnicity and racism.

Reporting on sex and gender	Sex is reported in the manuscript in the Sample description, Methods section, page 16. Only males were included in the current study to decrease heterogeneity given the limited sample size. Future work will increase sample size and will add females in addition to males.
Reporting on race, ethnicity, or other socially relevant groupings	Ethnicity is reported in the manuscript in the Sample description, Methods section, page 16. We have included European Americans and African Americans.
Population characteristics	Covariates included age, ancestry, cigarette smoking, and posttraumatic stress disorder diagnosis. THis is described in the Differential analysis of methylation and hydroxymethylation, Methods section, page 18, and in the Co-methylation analysis, Methods section, page 19. Additional population characteristics (e.g., cause of death, toxicology-related information) is described in Sample description, Methods section, page 16 and included in Table 1.
Recruitment	Postmortem human brain specimens were obtained from the National Post-traumatic Stress Disorder (PTSD) Brain Bank (NPBB), a brain tissue repository at the US Department of Veterans Affairs. This is described in Sample description, Methods section, page 16.
Ethics oversight	This study was approved by the Institutional Review Board Committees of the Department of Veterans Affairs and Yale School of Medicine. This is described in Samples description, Methods section, page 16.

Note that full information on the approval of the study protocol must also be provided in the manuscript.

Field-specific reporting

Please select the one below that is the best fit for your research. If you are not sure, read the appropriate sections before making your selection.

☒ Life sciences ☐ Behavioural & social sciences ☐ Ecological, evolutionary & environmental sciences

For a reference copy of the document with all sections, see nature.com/documents/nr-reporting-summary-flat.pdf

Life sciences study design

All studies must disclose on these points even when the disclosure is negative.

Sample size	Postmortem human brain specimens were obtained from the National Post-Traumatic Stress Disorder (PTSD) Brain Bank (NPBB) (Friedman et al. 2017), a brain tissue repository in the U.S. Department of Veterans Affairs (VA). In addition, brain tissue samples were collected from the orbitofrontal cortex (OFC; Brodmann Area 11). The cause of death in the non-OUD group included natural causes, suicide, accident causes, and alcohol and drug intoxication. None of the individuals in the non-OUD group had a history of OUD diagnosis. All individuals in the OUD group were diagnosed with OUD and died from drug and/or alcohol intoxication, including, but not limited to, opioids. No power calculations were performed, given the limited sample size in the postmortem brain. However, our sample size is comparable to similar work (Kozlenkov et al. 2017) using human postmortem brain tissue in the context of substance use disorder, including opioid use disorder. This is described in the discussion section, page 15.
Data exclusions	For this work, only men samples were included to decrease heterogeneity. Two samples were excluded in the QC analyses.
Replication	We did not perform an independent replication analysis in this paper due to the absence of similar cohorts and data. However, we compared our findings to a similar study by Kozlenkov et al. (2017) that analyzes DNA methylation of heroin abuse in human postmortem orbitofrontal cortex, same brain region as in the current study. We also conducted a multi-tissue comparison with additional DNA methylation studies (six) in opioid-related traits in other brain regions (2021) and tissues (2020-2023). We observed replication and overlap of our findings. This is described in the Results and Discussion.
Randomization	Experimental groups were matched by age of death, postmortem interval, ancestry, cigarette smoking, and alcohol dependence.
Blinding	Blinding was not relevant to our study. This is a case-control study design in human postmortem brain tissue.

Reporting for specific materials, systems and methods

We require information from authors about some types of materials, experimental systems and methods used in many studies. Here, indicate whether each material, system or method listed is relevant to your study. If you are not sure if a list item applies to your research, read the appropriate section before selecting a response.

Materials & experimental systems

n/a	Involved in the study
☐	☒ Antibodies
☒	☐ Eukaryotic cell lines
☒	☐ Palaeontology and archaeology
☒	☐ Animals and other organisms
☒	☐ Clinical data
☒	☐ Dual use research of concern
☒	☐ Plants

Methods

n/a	Involved in the study
☒	☐ ChIP-seq
☐	☒ Flow cytometry
☒	☐ MRI-based neuroimaging

Antibodies

Antibodies used	Antibodies used included Anti-NeuN-PE (Millipore-Sigma; FCMAB317PE), and DAPI. This is described in the Fluorescence-Activated Nuclei Sorting Methods section.
Validation	According to the manufacturer's description, the Anti-NeuN-PE Antibody is validated for use in FC for the detection of NeuN (Millipore-Sigma).

Flow Cytometry

Plots

Confirm that:

☒ The axis labels state the marker and fluorochrome used (e.g. CD4-FITC).

☒ The axis scales are clearly visible. Include numbers along axes only for bottom left plot of group (a 'group' is an analysis of identical markers).

☒ All plots are contour plots with outliers or pseudocolor plots.

☒ A numerical value for number of cells or percentage (with statistics) is provided.

Methodology

Sample preparation	Samples were lysed in homogenization buffer, filtered, loaded onto sucrose cushion, and ultracentrifuges. Nuclei were then underwent fluorescence-activated cell sorting (FANS) carried out at the Icahn School of Medicine Flow cytometry CoRE. DNA from neuronal nuclei was then extracted and sequenced using reduced representation oxidative bisulfite sequencing at the Weill Cornell Epigenomics Core.
Instrument	Illumina NovaSeq6000 system.
Software	BD 5-laser cell sorting system.
Cell population abundance	After FANS, sorted nuclei were processed to the DNAeasy Blood and Tissue Kit and eluted samples were further concentrated to 20ul final volume with the Zymo Genomic DNA Clean and Concentrator-10 kit.
Gating strategy	The gating strategy was conducted following the BD 5-laser cell sorting system conducted at the Icahn School of Medicine Flow Cytometry CoRE.

☒ Tick this box to confirm that a figure exemplifying the gating strategy is provided in the Supplementary Information.
